# Capnodynamics – noninvasive cardiac output and mixed venous oxygen saturation monitoring in children

**DOI:** 10.3389/fped.2023.1111270

**Published:** 2023-02-03

**Authors:** Jacob Karlsson, Per-Arne Lönnqvist

**Affiliations:** ^1^ Dept of Physiology & Pharmacology, Section of Anaesthesiology and Intensive Care, Karolinska University Hospital, Stockholm, Sweden; ^2^ Paediatric Perioperative Medicine & Intensive Care, Karolinska University Hospital, Stockholm, Sweden

**Keywords:** cardiac output (CO) monitoring, capnography, pediatric, mixed venous blood oxygen saturation, monitoring

## Abstract

Hemodynamic monitoring in children is challenging for many reasons. Technical limitations in combination with insufficient validation against reference methods, makes reliable monitoring systems difficult to establish. Since recent studies have highlighted perioperative cardiovascular stability as an important factor for patient outcome in pediatrics, the need for accurate hemodynamic monitoring methods in children is obvious. The development of mathematical processing of fast response mainstream capnography signals, has allowed for the development of capnodynamic hemodynamic monitoring. By inducing small changes in ventilation in intubated and mechanically ventilated patients, fluctuations in alveolar carbon dioxide are created. The subsequent changes in carbon dioxide elimination can be used to calculate the blood flow participating in gas exchange, i.e., effective pulmonary blood flow which equals the non-shunted pulmonary blood flow. Cardiac output can then be estimated and continuously monitored in a breath-by-breath fashion without the need for additional equipment, training, or calibration. In addition, the method allows for mixed venous oxygen saturation (SvO_2_) monitoring, without pulmonary artery catheterization. The current review will discuss the capnodyamic method and its application and limitation as well as future potential development and functions in pediatric patients.

## Introduction

One of the main goals when caring for critically ill children, whether in the intensive care or operating room setting, is ensuring adequate oxygen delivery (1, 2). End organ hypoperfusion is a known contributing factor to poor outcome and therefore several attempts at monitoring end organ perfusion, as well as developing associated treatment strategies, have been made (3, 4). However, the success of such efforts is dependent on reliable monitoring of both macro and microcirculation, something that has been hard to establish in pediatrics for several reasons. Technical challenges due to small size and unacceptably high risk (e.g., pulmonary artery catheter) or need for specific expertise and training (e.g., cardiac echo or laser Doppler flow measurements) have so far limited the access to advanced hemodynamic monitoring in children. The need for safe, reliable, and technically easy monitoring in children is therefore obvious.

Recent publications have highlighted the link between blood pressure and blood flow (e.g., cardiac output, CO) in children as relatively weak (5). The relation between pressure and flow is further complicated by lack of definition of “normal” blood pressure and the presence of cardiac shunts with imbalance between systemic and pulmonary blood flow. Several attempts have been made to defined normal values of blood pressure (6, 7). Even if such reference values may be useful, they still tell us very little about oxygen delivery and end organ perfusion. To overcome this, our research group developed the capnodynamic method, based on the differential Fick's principle (8). By inducing small, controlled changes in respiratory pattern in mechanically ventilated patients, variations in alveolar CO_2_ are created. Subsequent mathematical handling of mainstream CO_2_ recordings of these variations can then be used to give an extensive view of the patient's circulatory status. The method allows for continuous monitoring of effective pulmonary blood flow (e.g., the blood flow that participates in gas exchange, thus excluding shunted blood flow) (9). In addition, mixed venous oxygen saturation (SvO_2_) can be estimated without the need for pulmonary artery catheter (10). By simultaneous measurement of cardiac output, blood pressure and mixed venous oxygen saturation, capnodynamics narrows the gap between macro- (e.g., pressure and flow) and micro-circulation (e.g., SvO_2_) monitoring. By adding more novel concepts of microcirculatory assessment such as the pulmonary artery to systemic arterial carbon CO_2_ content difference, the method may be expanded to give an even more widespread picture of the circulation in the future (11). This review will summarize the background of the capnodynamic method, its validation in children, and its role in relating flow to pressure in clinical studies and practice. In addition, limitations with the method as well as future potential applications in microcirculatory monitoring will be discussed.

## The capnodynamic method

The basic concept behind the capnodynamic method is the differential Fick principle (8). The principle is based on Adolf Fick's classic gold standard for cardiac output monitoring (12). The concept is based on conservation of mass over the pulmonary circulation where the uptake of oxygen in the alveoli must equal the difference between arterial and pulmonary mixed venous oxygen content as shown in the equation below:

(1) $$\mathrm{CO}=\frac{VO_{2}}{\left(\mathrm{Ca}O_{2}-\mathrm{Cv}O_{2}\right)}$$

Equation 1: CO; Cardiac Output, VO_2_; oxygen consumption, CaO_2_; arterial oxygen content, CvO_2_; mixed venous oxygen content.

**Figure 1:**
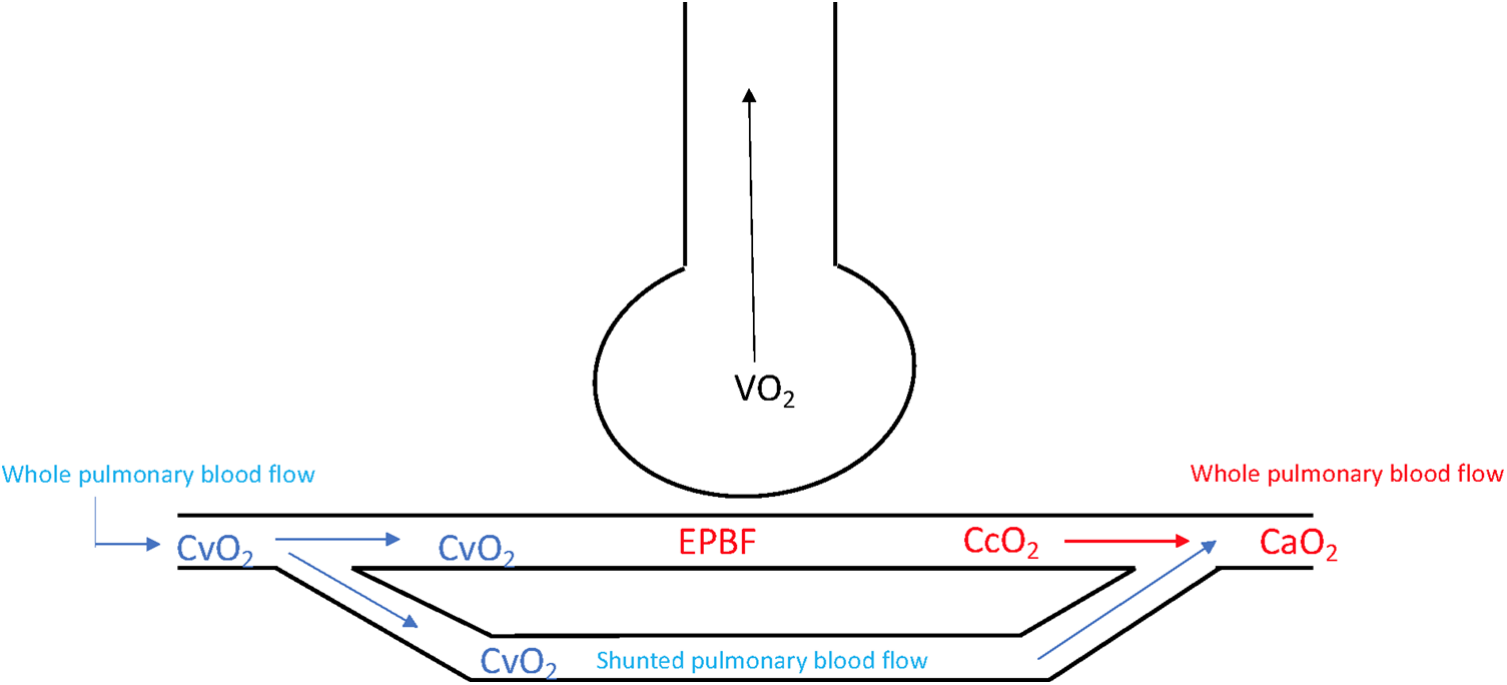
A schematic presentation of a perfused alveolus, showing the difference between whole pulmonary and non-shunted blood pulmonary blood flow. The capnodynamic method estimates the Effective Pulmonary Blood Flow (EPBF, i.e., non-shunted pulmonary blood flow) which in the absences of large pulmonary shunts equals whole pulmonary blood flow (i.e., the sum of shunted and non-shunted pulmonary blood flow). Note that the mixed venous oxygen content is the same regardless of using Fick's equation for non-shunted (VO_2_/(CcO_2_-CvO_2_) or whole pulmonary blood flow (VO_2 _= CaO_2_-CvO_2_) which is why the capnodynamic SvO_2_ calculation is less affected by shunt ratio since than when capnodynamics is used for estimation of cardiac output. EPBF; Effective Pulmonary Blood Flow, VO_2_; oxygen consumption, CaO_2_; arterial oxygen content, CvO_2_; mixed venous oxygen content; CcCO_2_; pulmonary end capillary carbon dioxide content.

The original Fick's principle estimates the whole pulmonary blood flow, i.e., shunted, and non-shunted flow. Provided that no major pulmonary shunt exists, the arterial oxygen content in Equation 1 can however be substituted for pulmonary end capillary oxygen content (CcO_2_). Figure 1 below illustrates the difference between shunted and non-shunted pulmonary blood flow. Classic Fick's principle for CO estimation, as shown in Equation 1, is known as direct Fick and involves measurement of oxygen uptake and pulmonary mixed venous and arterial oxygen content, a task requiring cumbersome equipment such as Douglas bag estimation of VO_2_ and pulmonary artery catheterization (13). For comparison, indirect Fick is based on the same principle but utilizes a calculated estimation of VO_2_. By substituting O_2_ with CO_2_, the direct Fick method can be simplified since measurements of CO_2_ are easier to obtain. Equation 1 can then be rearranged as follows:

(2) $$\mathrm{CO}=\frac{\mathrm{VC}O_{2}}{\left(\mathrm{CvC}O_{2}-\mathrm{CcC}O_{2}\right)}$$

Equation 2: CO; Cardiac Output, VCO_2;_ carbon dioxide elimination, CvCO_2_; mixed venous carbon dioxide content, CcCO_2_; pulmonary end capillary carbon dioxide content.

This concept, substituting O_2_ by CO_2_ in Fick's equation, is known as the direct CO_2_ Fick method for cardiac output estimation and forms the theoretical basis for the capnodynamic method. VCO_2_ can be measured using volumetric capnography in a relatively straightforward way (14). However, the method still requires invasive pulmonary artery catheterization to obtain mixed venous oxygen content and, in addition, a way of estimating CcCO_2_. The issue with estimating CcCO_2_ was overcome when Capek et al. showed that CcCO_2_ can be accurately measured by analysis of exhaled CO_2_ fraction in combination with the CO_2_ dissociation curve (15). Using this approach, CcCO_2_ and VCO_2_ can be estimated continuously with high precision and applied in Equation 2. However, the equation still requires the input of mixed venous CO_2_ content thus necessitating a pulmonary artery catheter. In 1980 Gedeon et al. solved this problem by introducing the differential Fick equation (8). In the differential Fick equation, VCO_2_ and CcCO_2_ estimations are made at two different time points separated by a small change in alveolar ventilation. This small change in alveolar ventilation creates perturbations in alveolar CO_2_ during which the mixed venous CO_2_ content can be considered stable. This creates two equations for CO, one at steady state before change in alveolar CO_2_ and one after change in alveolar CO_2_. Provided that the mixed venous CO_2_ content is constant for the duration of the changes, CvCO_2_ can be shortened from the equation. Small, induced variations in alveolar CO_2_ can then be used in combination with continuous analysis of VCO_2_ and CcCO_2_ to estimate the effective pulmonary blood flow (EPBF). EPBF represents the blood flow that participates in gas exchange (i.e., non-shunted pulmonary blood flow) which in the absence of major pulmonary shunts equals systemic cardiac output (16).

Assumption of stable mixed venous oxygen content during the changes in alveolar ventilation is a prerequisite of the capnodynamic method. If the variations in alveolar CO_2_ are small, the subsequent increase in alveolar CO_2_ will not be reflected in the mixed venous CO_2_ content. This is because the net flux of CO_2_ reaching the pulmonary artery is a mixture of CO_2_ from all compartments in the body. The small changes in alveolar CO_2_ generated by the ventilatory pattern will cause variations in end capillary CO_2_ but these variations are “diluted” by the returning venous systemic blood where blood from different compartments with different circulatory time constants mixes (17). Therefore, if the variations in alveolar CO_2_ are optimal in magnitude and not too long, CvCO_2_ can be regarded as constant throughout the respiratory cycle. The changes in alveolar CO_2_ with the breathing pattern in combination with an illustration of stable CvCO_2_ and mixed venous blood flow are shown in a video (supplementary data 1).

## The capnodynamic equation

**Figure 2:**
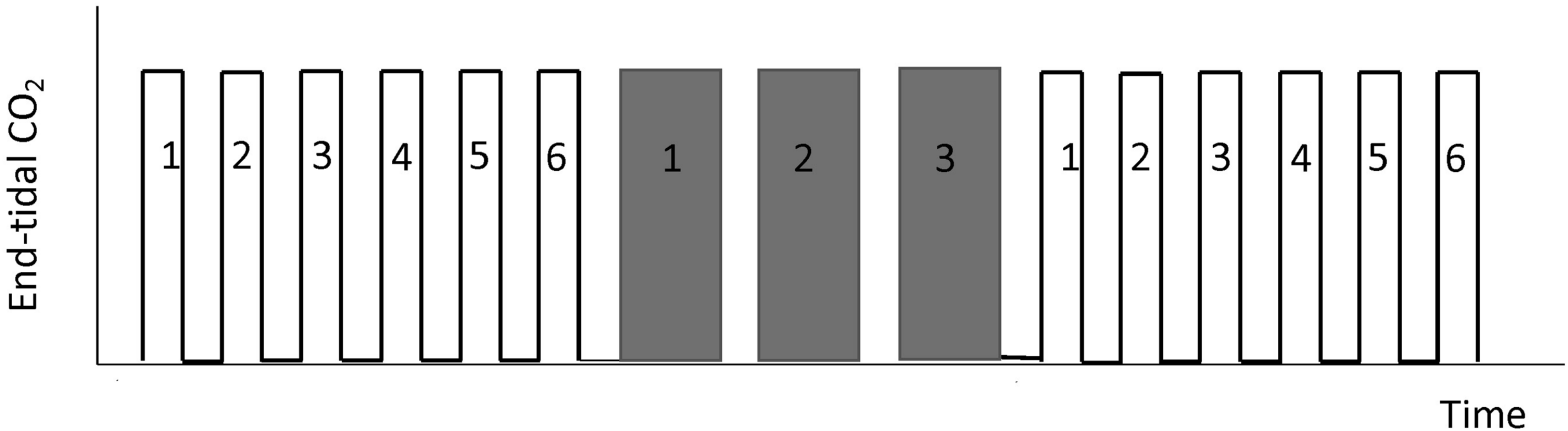
A schematic pattern of time-based capnography demonstrating the capnodynamic breathing cycle with 6 breaths with I:E ratio of 1:2 followed by 3 breaths with an expiratory pause. The variations in alveolar and end-tidal CO_2_ provides the data used in the capnodynamic Equation 3.

The capnodynamic method uses small changes in inspiratory to expiratory time ratio (I:E) to create the variations in alveolar ventilation described above. In practice, six breaths with normal I:E ratio are followed by three breaths with an expiratory pause (usually 1–2s) creating a characteristic breathing pattern shown in Figure 2. This leads to a functional decrease in respiratory rate and variations in alveolar CO_2_ of approximately 0.5–1 kPa between breaths with normal and prolonged I:E ratio. The continuous respiratory pattern is preprogrammed in a standard ventilator (Servo I, Maquet Critical Care, Solna. Sweden) and has under normal circumstances no impact on the subjects oxygenation, only on the alveolar CO_2_ fraction. Originally, the changes in I:E ratio were induced by inspiratory pauses whereas updated capnodynamic versions uses expiratory pauses. Expiratory pauses have been shown to improve the agreement between capnodynamic CO and established CO reference methods, when compared to inspiratory pauses (18).

The current capnodynamic equation used for continuous assessment of EPBF is shown and explained below (19).


(3) $$\mathrm{EEL}V_{CO_{2}}\cdot \left(\mathrm{FA}\;CO_{2}n-\mathrm{FAC}O_{2}n-1\right)=\mathrm{EPBF}\;\cdot \;\Delta t\;n\;\cdot \;\left(\mathrm{CvC}O_{2}-\mathrm{CcC}O_{2}n\right)-\mathrm{VTC}O_{2}n$$

Equation 3: FACO_2_, alveolar CO_2_ fraction; EELV_CO2_, End Expiratory Lung Volume CO_2_ (liter): volume containing CO_2_ at the end of expiration; EPBF, effective pulmonary blood flow (liter min^−1^); *n*, current breath; *n*-1, previous breath; C_v_CO_2_, venous carbon dioxide content (litergas literblood^−1^) CcCO_2n_, lung capillary CO_2_ content (calculated from FACO_2_); VTCO_2_n, volume (liter) of CO_2_ eliminated by the current, nth, breath; *Δ* tn, current breath cycle time (min).

With the capodynamic breathing pattern, each breath produces a single equation based on the molar balance for CO_2_ over the lung circulation as described above in Equation 3 (18). One series of six normal breaths and three breaths with expiratory pause, i.e., 9 breaths, thus form 9 separate equations. For each equation, EPBF, EELVCO_2_ and CvCO_2_ are unknown variables. The remaining variables are continuously measured *via* mainstream and volumetric capnography in a breath-by-breath fashion. By solving the over-determined system for the 9 separate equations using the least square error method, the equation system can be solved and EPBF, EELVCO_2_ and CvCO_2_ calculated.

Since the equation system is solved and updated with each breath, the capnodynamic method gives information on EPBF (and the other parameters) based on the last 9 breaths. The response time for change is consequently a function of the duration of 9 breaths, which depends on the respiratory rate. If a child for example has a respiratory rate of 30 breaths per minutes (i.e., 2 s per breath), the response time for change in EPBF will be 9 × 2 s i.e., 18 s.

In addition to EPBF and CvCO_2_, the equation also calculates EELVCO_2_ (end-expiratory lung volume CO_2_) a variable that represents the volume of the lung containing CO_2_ at the end of expiration. EELVCO_2_ has been shown to be closely related to functional residual capacity (FRC) in several experimental and clinical studies and is therefore of interest from a ventilatory and heart-lung interaction perspective (20–22).

All calculations are continuously checked against an ideal one-lung compartment model, a theoretical mathematical model of how alveolar CO_2_ varies with the breathing pattern in an open lung with ideal gas exchange. If the difference between the modelled data and the measured/calculated data is too wide, the system will recognize this as unreliable and no EPBF, EELVCO_2_ and CvCO_2_ variables are presented to eliminate the risk of displaying false numbers.

The variations in alveolar CO_2_ induced by the changes in I:E ratio used in the capnodynamic method, can also be induced using other strategies. An example is partial CO_2_ rebreathing by adding apparatus dead space as described by Capek and Roy (15). A commercially available method, NICO (NICO Respironics, Murrysville PA) has been developed where also shunt fraction is compensated for with Nunn's iso shunt fraction plots (23, 24). Clinical studies comparing partial CO_2_ rebreathing with pulmonary artery thermodilution in adults, have shown acceptable agreement (25). Partial rebreathing using dead space have also been evaluated in children, with acceptable agreement against reference methods (26). The system is However currently not validated for patients with tidal volumes of <300 ml or in subjects <15 kg.

## Validation of the capnodynamic method

The capnodynamic method for continuous cardiac output assessment has been extensively validated against a large variety of reference methods in both experimental and clinical studies (9, 27–35). Like most method comparison studies, the initial focus has been on relating the methods accuracy against absolute values obtained with “gold standard” reference techniques and its ability to detect change (trending ability). Once the method is accurately tested against reference methods, its capacity to function as a hemodynamic monitoring tool in practice can then be investigated.

Validation studies have also been the focus in the pediatric population, where capnodynamics has been tested in piglet models and in clinical scenarios against established reference methods (e.g., transpulmonary flow probe, transpulmonary thermodilution and direct CO_2_ Fick and suprasternal transesophageal Doppler) (9, 27–35).

Most of the experimental and clinical validation studies in children have shown good agreement against gold standard with bias and limits of agreement within acceptable predefined limits.

For both experimental and clinical adult studies, the impact of pulmonary shunt seems to be a determining factor for accuracy against gold standard whereas trending ability is generally well preserved even in the presence of larger shunt fractions (27). The impact of lung shunt on accuracy also seems to be reversible and respond to lung recruitment which has been shown in both experimental and clinical studies (27, 30, 35).

In addition, major fluctuations in CvCO_2_, experimentally induced in an animal model of regional hypoperfusion *via* descending aortic balloon inflation for 30 min, temporarily affects the accuracy of the method even if these changes are transient (e.g., restored within 5 min after reperfusion) and trending ability still preserved (29).

Like most cardiac output monitoring methods, accuracy is thus affected at extremes of physiology, which is often when a monitoring method is needed most. However, this largely appears to be a transient and reversible phenomenon even if it is important to keep this potential limitation in mind.

**Figure 3:**
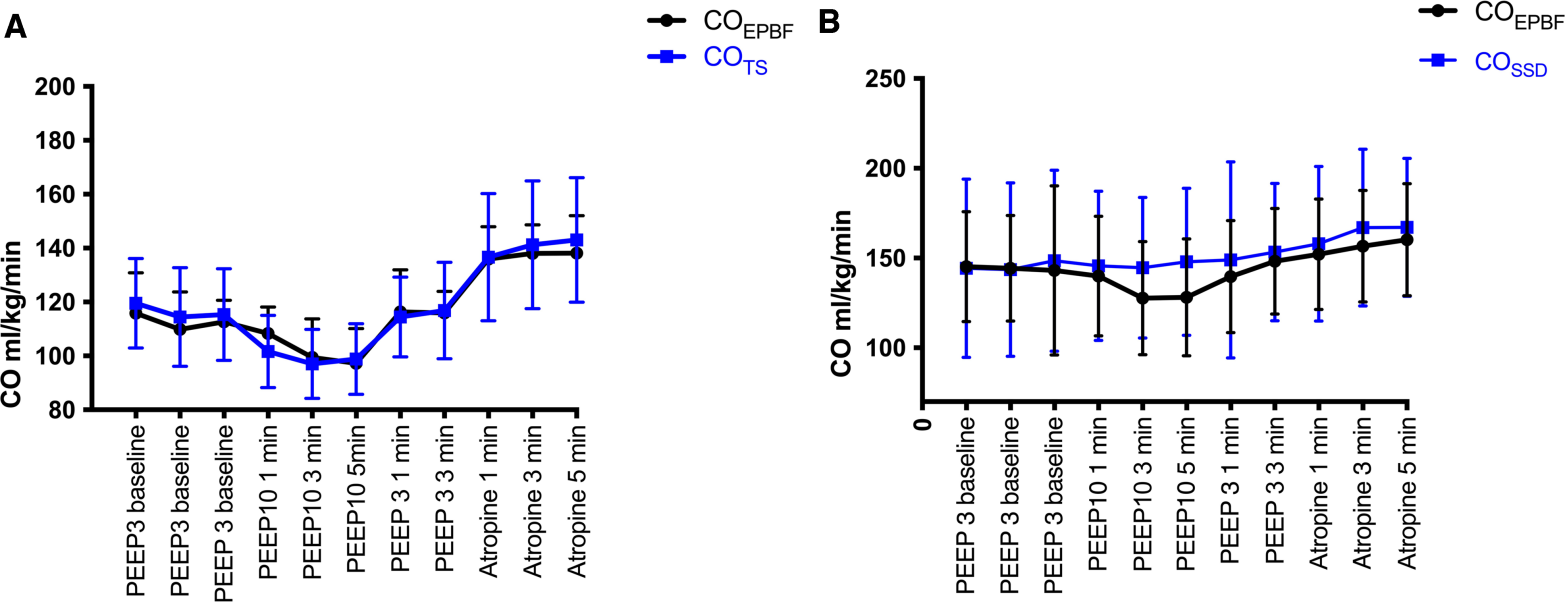
Event plot illustrating the agreement between capnodynamic cardiac output (CO_EPBF_) and the gold standard transpulmonary artery flow probe in the experimental study (**A**) and against 2D pulsed suprasternal Doppler (CO_SSD_) in the clinical study (**B**), in response to PEEP and atropine exposure. Values are mean ± SD (*N* = 9 experimental study, *N* = 15 clinical study). The relatively large spread in CO in the clinical study is caused by age dependent variations in cardiac output (cf. the minimal spread in the homogenous animal population). CO_EPBF_, Cardiac output Effective Pulmonary Blood Flow; PEEP, Positive End-Expiratory Pressure. Modified from reference (27).

The first clinical application of the capnodynamic method was made by our research group in 2018 when capnodynamically derived EPBF was compared against 2 D suprasternal Doppler measurement of CO in anesthetized children undergoing cleft palate repair (15 patients median age 8.5 months, median weight 8.3 kg) (33). In a series of PEEP challenges, intended to reduce preload and decrease CO, as well as intravenous atropine to increase CO, EPBF performed well against the comparison method (suprasternal 2D Doppler operated by experienced pediatric cardiologist). Interestingly, PEEP and atropine induced CO changes were not easily identified by suprasternal Doppler. For this reason, the study was replicated in piglets in an experimental setting to confirm the CO response to PEEP and atropine, using 10 piglets exposed to the exact same hemodynamic challenges as in the clinical study. This time the invasive gold standard, transpulmonary artery flow probe, was used as reference. The agreement of absolute values showed a mean percentage error of 31%, indicating acceptable agreement. In addition, the capnodynamic method had a 100% concordance rate with the gold standard indicating its ability to detect the same changes in CO as the reference method in 100% of the cases. The results points towards a more reliable performance of the capnodynamic method in assessing CO and change in CO than suprasternal Doppler performed by an experienced cardiac sonographer. Figure 3 below shows a time plot of the capnodynamic method against CO_TS_ (A) in the experimental study and against suprasternal 2D Doppler in the clinical study (B) in response to PEEP exposure and atropine.

**Figure 4:**
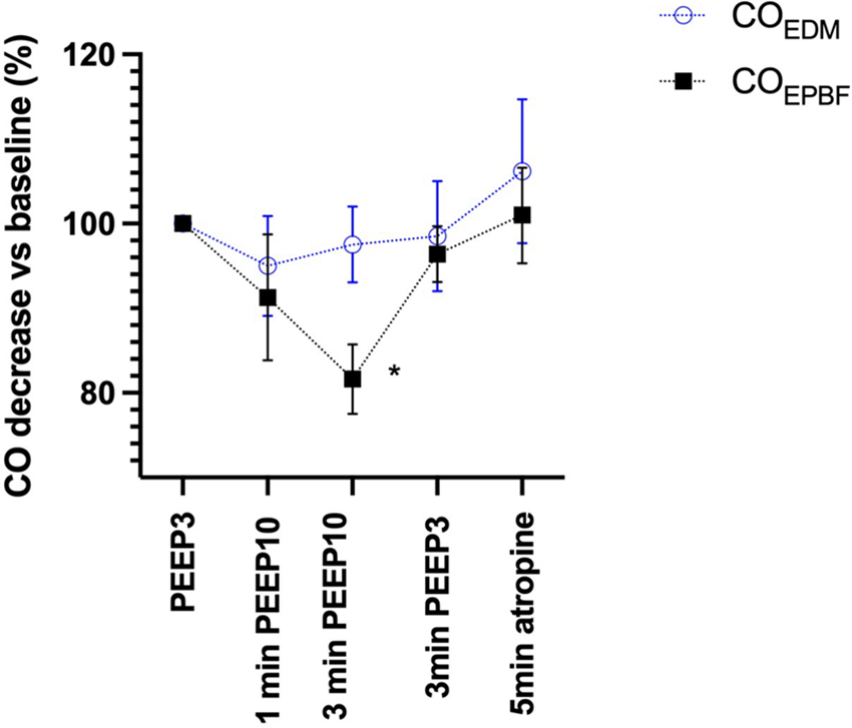
Event plot illustrating relative changes in esophageal Doppler and CO_EPBF_ cardiac output measurements. CO_EPBF_ identifies the PEEP induced cardiac output decrease which is not detected up by the esophageal Doppler technique. Values are mean (95%CI), *N* = 15. *P < 0.05 vs. baseline. CO_EPBF_, Cardiac output Effective Pulmonary Blood Flow; PEEP, Positive End-Expiratory Pressure; CO_EDM_, Cardiac Output Esophageal Doppler Monitor. Modified from reference (25).

An additional clinical validation study in infants has been performed, using transesophageal Doppler (CardioQ-ODM+®, Deltex Medical Ltd, Chichester, United Kingdom) as reference. 15 infants (median age and weight 8 months and 9 kg respectively, smallest 3.7 kg) were exposed to PEEP changes, intended to create variations in CO (31). Esophageal Doppler failed to detect the induced changes in CO, which were identified by capnodynamics. Equally important, noninvasive blood pressure failed to detect these changes. In addition to validating capnodynamics as tool for detecting minor hemodynamic changes, the study therefore also demonstrates the important difference between pressure and flow. In spite an 18% reduction in CO seen after PEEP exposure, NIBP remained stable thus highlighting the limitations of using NIBP as a surrogate for flow and oxygen delivery (31). Figure 4 below shows the changes in CO assessed with both EPBF and transesophageal Doppler from this study.

In addition to the clinical and experimental validation studies in children mentioned above, capnodynamics has also been validated in experimental pediatric models of pulmonary hypertension (PHT) (32). In a hypoxic gas mixture model of PHT (causing selective pulmonary vasoconstriction) and subsequent inhaled nitric oxide (iNO) administration, capnodynamics performed well against transpulmonary artery flow probe and direct CO_2_ Fick. This is an important finding, particularly when transitioning the capnodynamic method from the OR into the pediatric intensive care setting where pulmonary hypertension and iNO are relatively common conditions, who's clinical course may be challenging to follow without solid knowledge in echocardiography.

## Cardiac anesthesia

One of the potentially most useful areas in for the capnodynamic method is in congenital cardiac surgery. The method is currently being tested in children with congenital heart disease with the intention to establish capnodynamics in the cardiac operating room in the future. So far, the method has been tested in the adult cardiac surgical setting in one study using transpulmonary thermodilution as reference, showing a mean percentage error of 31% intraoperatively (34). Even if these results are promising, the situation may be very different when examining children with congenital heart disease, shunts, and complete mixing of systemic venous return and pulmonary venous return blood (e.g., single ventricle physiology) where EPBF may not represent systemic blood flow. This relation is currently investigated in clinical studies.

The same is true for the capnodynamic performance in the intensive care setting where so far only one adult study exists (36). It remains to be investigated how EPBF performs in these situations where conditions for open lung ventilation may be more challenging to obtain than in the operating room.

Even if the current validation for capnodynamics points towards its usefulness in children, several situations characteristic of the pediatric setting remains to be investigated. The impact of cardiac shunts is still not clear as mentioned above and major compromise in lung mechanics may also provide a limitation. In addition, it's performance in neonates and premature children has not been investigated. Apart from validation in various clinical contexts, the need for alternative validation methods is also a challenge. Traditional gold standard techniques such as pulmonary artery thermodilution are very hard to obtain in small children due to equipment limitations. Furthermore, the reference method ideally needs a response time within the same magnitude as the tested method, to accurately evaluate ability to detect changes (37). The difficulty with obtaining reliable reference methods in small children reflects the overall uncertainty associated with almost all hemodynamic monitoring. Since no true gold standard exists for monitoring CO and oxygen delivery (after all, if a gold standard does exist, what is that gold standard validated against?), it is perhaps equally important to monitor the net result of the oxygen delivery, i.e., the balance against oxygen consumption. One way of doing this is to assess the mixed venous oxygen saturation, a parameter reflecting the net oxygen consumption regardless flow or blood pressure thereby partially bypassing the issue with precise measurement of flow or using blood pressure as a surrogate for blood flow and oxygen delivery. Thanks to recent development of the capnodynamic method, this has been made possible by simple mathematical modeling. The concept of non-invasive mixed venous oxygen assessment and its limitations is discussed below.

## Noninvasive mixed venous oxygen saturation

Capnodynamic assessment of CO based on Fick's principle with CO_2_, enables the classic Fick equation for non-shunted blood flow [CO = VO_2_/(CcO_2_-CvO_2_)] to be solved for CvO_2_ if oxygen consumption (VO_2_) is known or can be estimated. The CO calculated form dynamic capnography (EPBF) should equal VO_2_/(CcO_2_-CvO_2_) as shown in the equation below (Equation 4).


(4) $$\mathrm{EPBF}\;\;=\frac{VO_{2}}{\left(\mathrm{Cc}O_{2}-\mathrm{Cv}O_{2}\right)}$$

Equation 4. EPBF, Effective Pulmonary Blood Flow; VO_2_; oxygen consumption, CcO_2_; pulmonary end capillary oxygen content, CvO_2_; mixed venous oxygen content.

The oxygen consumption VO_2_ in Equation 4 can be calculated *via* a measured VCO_2_ (obtained *via* volumetric capnography) and knowledge of the respiratory quotient (RQ) since RQ = VCO_2_/VO_2_ and thus VO_2 _= VCO_2_/RQ. RQ can be measured *via* direct methods or obtained *via* tabulated values. The CcO_2_ in Equation 4 above can be calculated using the alveolar gas equation and CvO_2_ can then be estimated since this in the only unknown variable (10). From the mixed venous oxygen content, the mixed venous oxygen saturation SvO_2_ can then be estimated *via* the equation for blood oxygen content.

**Figure 5:**
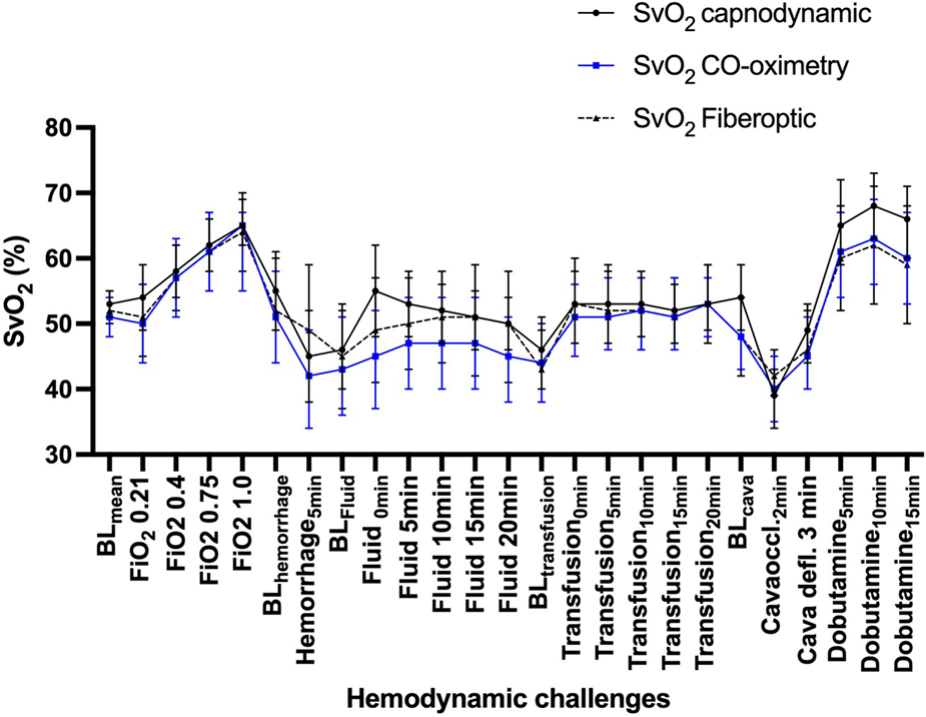
Event plot showing the variations in SvO2 for three different monitoring methods, CO-oximetry, fiber optic and capnodynamic SvO_2_ measurements. The x-axis shows a series of hemodynamic challenges involving exposure to variations in inspiratory oxygen fraction, hemorrhage, crystalloid and blood transfusion, inferior cava vein occlusion and dobutamine infusion. Capno-SvO2 showed comparable performance for absolute values compared to both CO-oximetry and fiber optic SvO_2_ and had a trending ability of 98%. Values are mean (95%CI), *N* = 11. Modified from reference (32).

By this combination of classic oxygen Fick and differential Fick for EPBF, SvO_2_ can be assessed in a breath-by-breath fashion without the need for pulmonary artery catheter (Capno-SvO_2_). The method has so far been validated in two pediatric experimental piglet models against gold standard blood gas CO-oximetry from mixed venous blood gases and against continuous fiber optic SvO_2_ measurement. The first study investigated Capno-SvO_2_ for its ability to detect minor to moderate PEEP and atropine-based changes in SvO_2_ (10). The overall bias was −1 percentage point (95% limits of agreement −13 to +11 percentage points) which indicates close agreement between Capno-SvO_2_ and gold standard CO-oximetry. More importantly, the trending ability was 100% for Capno-SvO_2_ compared to gold standard. To further challenge the method, an additional pediatric piglet model of major hemodynamic changes was used, using CO-oximetry and fiber optic SvO_2_ as references (38). 11 piglets were exposed to a series of variations in inspiratory oxygen fraction, hemorrhage, crystalloid and blood transfusion, inferior cava vein occlusion and dobutamine infusion. Capno-SvO_2_ showed overall comparable performance against CO-oximetry and fiberoptics indicating that the method is also reliable during major changes in oxygen delivery. Figure 5 below shows a time plot with Capno-SvO_2_ against the two reference methods, CO-oximetry and fiberoptic SvO_2_, during various hemodynamic challenges.

The application of continuous SvO_2_ estimation using Capno-SvO_2_ may be useful to determine overall balance between oxygen delivery and consumption in children. Even if true mixed venous oxygen saturation is hard to obtain, particularly in pediatrics where the use of pulmonary artery catheters is rare, the surrogate measure central venous oxygen (ScVO_2_) has been shown to be beneficial as a monitoring tool after for instance congenital heart surgery and to guide treatment of critically ill children (39, 40). The access to continuous noninvasive SvO_2_ assessment would therefore potentially be beneficial. It is however important to remember that the theory behind the capnodynamic technique is based on the blood flow in a structurally normal heart. A major challenge for both EPBF and Capno-SvO_2_ in the pediatric setting, is therefore to investigate their performance in the presence of intra- and extra-cardiac shunts, situations where the interpretation of CO-oximetry SvO_2_ is not always straightforward (41). In situations with intra-cardiac left to right shunting, such as for instance septal defects, the CO_2_ content in pulmonary arterial blood will be affected by mixing of pulmonary venous blood with low CO_2_ content and systemic venous return with higher CO_2_ content. Equally, in the presence of complete mixing in for instance hypoplastic left heart syndrome, the mixed blood reaching the pulmonary circulation does not reflect true systemic mixed venous oxygen content (41). It is in these situations that continuous assessment of SvO_2_ may be of particular interest and the performance of Capno-SvO_2_ and EPBF is therefore currently investigated in clinical studies.

## Limitations with capnodynamic monitoring

There are several limitations associated with the capnodynamic technique. The most important factor is that the method requires intubation and mechanical ventilation (17). Most ventilatory modes, e.g., pressure or volume ventilation, work but spontaneous or pressure supported ventilation is not possible using the method in its current form. In addition, the airway handling must be such that no leakage occurs around the endotracheal tube and alternative airway strategies such as laryngeal mask may therefore be inadequate.

The respiratory rate may also be a limiting factor since the principle with alternating breaths with various I:E relation affects the functional respiratory rate. In small subjects, such as neonates or premature babies, this may be a restricting factor even if the method has been used and validated in patients down to 3.7 kg infants (31). In addition, the response time of the mainstream capnography can also be a limiting factor in high respiratory rates (42). Validation of the method in small neonates and premature babies is further challenged by the lack of access to accurate reference methods in these age groups as previously discussed.

Form a circulatory point of view, the presence of large pulmonary shunts will also affect the accuracy of the method. Capnodynamic CO estimates the blood flow that participates in gas exchange and large pulmonary shunts will therefore affect the agreement of EPBF with methods measuring the entire CO (i.e., including shunted pulmonary blood flow). The assessment of Capno-SvO_2_ appears more robust in these situations and has been to show to perform well even with shunt levels of >20% (10). This can most likely be explained by the fact that mixed venous oxygen content is the same regardless of Fick's equation is used for arterial (i.e., whole pulmonary blood flow) or pulmonary end capillary oxygen content (i.e., non-shunted pulmonary blood flow) as can be seen from Equations 1, 4 and in Figure 1. From Figure 1 it is apparent that the mixed venous oxygen content is the same regardless of Fick's equation is used for shunted or whole (shunted and non-shunted) pulmonary blood flow. Nevertheless, adequate measures to ensure open lung conditions should be taken to optimize the methods accuracy, a strategy likely beneficial regardless of if the capnodynamic method is used or not (43).

## Veno-arterial CO_2_ gap-a potential future application

Even if a normal SvO_2_ provides a good indicator of whole-body circulation, peripheral and organ specific hypoperfusion may still exist that is not detected by variations in SvO_2_. To better assess local disturbances in oxygen delivery, recent development has focused on assessing the CO_2_ gap between pulmonary venous and arterial blood, the “veno-arterial CO_2_ gap”. A large gap may indicate an imbalance between cardiac output and peripheral CO_2_ production (44). This has been shown to reflect microcirculatory changes assessed by side stream dark field imaging of capillary flow in early phases of shock in adults and aid in identifying anaerobic metabolism in septic shock even in the presence of normal SvO_2_ (45). Pediatric studies looking at outcome after neonatal and pediatric cardiac surgery have linked veno-arterial CO_2_ gap to low cardiac output state even if the value of this parameter in critically ill children has been conflicting (46–49). The method requires pulmonary arterial (or surrogate central venous) and systemic arterial blood sampling to calculate the gap. Since the capnodynamic method estimates CO based on VCO_2_/(CvCO_2_-CaCO_2_) the gap CvCO_2_-CaCO_2_ can be calculated (gap = VCO_2_/EPBF) and potentially used to assess changes in microcirculation. Experimental studies of this relation are currently being undertaken and may provide an additional application of the capnodynamic method in children.

## Summary

Dynamic capnography appears to be a relatively accurate and easily accessible way of continuous CO and SvO_2_ monitoring in children with the advantage of not requiring any additional equipment, calibrations, or expertise. The ability to obtain both CO and SvO_2_ gives a comprehensive view of the hemodynamic status of the patient. Even if blood pressure can be accurately measured in children, the relation to blood flow and oxygen delivery is largely unknown. Simultaneous assessment of pressure and flow may contribute a better understanding of the hemodynamic shifts and help define what a “normal” blood pressure is in pediatric anesthesia and intensive care. In combination with noninvasive or invasive blood pressure recordings, dynamic capnography may therefore help bridging the gap between blood pressure and blood flow in pediatrics. In addition, novel applications such as Capno-SvO_2_ and non-invasive veno-arterial CO_2_ gradient, may help to link changes in routine monitoring of blood pressure, to changes in flow and micro-circulation even if this needs to be validated and investigated in further studies.

To be fully established in the pediatric setting, the next natural step for capnodynamics is now to be further tested in clinical situations.
